# An Insight into the Relationships between Hepcidin, Anemia, Infections and Inflammatory Cytokines in Pediatric Refugees: A Cross-Sectional Study

**DOI:** 10.1371/journal.pone.0004030

**Published:** 2008-12-24

**Authors:** Sarah Cherian, David A. Forbes, Angus G. Cook, Frank M. Sanfilippo, Erwin H. Kemna, Dorine W. Swinkels, David P. Burgner

**Affiliations:** 1 School of Pediatrics and Child Health, University of Western Australia, Perth, Western Australia, Australia; 2 Pediatric Infectious Diseases and Refugee Health, Princess Margaret Hospital for Children, Perth, Western Australia, Australia; 3 School of Population Health, University of Western Australia, Perth, Western Australia, Australia; 4 Pediatric Gastroenterology, Princess Margaret Hospital for Children, Perth, Western Australia, Australia; 5 Department of Clinical Chemistry, Radboud University Nijmegen Medical Centre, Nijmegen, The Netherlands; Universidad Nacional Mayor de San Marcos, Peru

## Abstract

**Background:**

Hepcidin, a key regulator of iron homeostasis, is increased in response to inflammation and some infections, but the *in vivo* role of hepcidin, particularly in children with iron deficiency anemia (IDA) is unclear. We investigated the relationships between hepcidin, cytokines and iron status in a pediatric population with a high prevalence of both anemia and co-morbid infections.

**Methodology/Principal Findings:**

African refugee children <16 years were consecutively recruited at the initial post-resettlement health check with 181 children meeting inclusion criteria. Data on hematological parameters, cytokine levels and co-morbid infections (*Helicobacter pylori*, helminth and malaria) were obtained and urinary hepcidin assays performed. The primary outcome measure was urinary hepcidin levels in children with and without iron deficiency (ID) and/or ID anaemia (IDA). The secondary outcome measures included were the relationship between co-morbid infections and (i) ID and IDA, (ii) urinary hepcidin levels and (iii) cytokine levels. IDA was present in 25/181 (13.8%). Children with IDA had significantly lower hepcidin levels (IDA median hepcidin 0.14 nmol/mmol Cr (interquartile range 0.05–0.061) versus non-IDA 2.96 nmol/mmol Cr, (IQR 0.95–6.72), p<0.001). Hemoglobin, log-ferritin, iron, mean cell volume (MCV) and transferrin saturation were positively associated with log-hepcidin levels (log-ferritin beta coefficient (β): 1.30, 95% CI 1.02 to 1.57) and transferrin was inversely associated (β: −0.12, 95% CI −0.15 to −0.08). Cytokine levels (including IL-6) and co-morbid infections were not associated with IDA or hepcidin levels.

**Conclusions/Significance:**

This is the largest pediatric study of the *in vivo* associations between hepcidin, iron status and cytokines. Gastro-intestinal infections (*H. pylori* and helminths) did not elevate urinary hepcidin or IL-6 levels in refugee children, nor were they associated with IDA. Longitudinal and mechanistic studies of IDA will further elucidate the role of hepcidin in paediatric iron regulation.

## Introduction

Iron deficiency anemia (IDA) is one of the commonest nutritional deficiencies globally, with young children, women of childbearing age and pregnant women at highest risk [1]. Adequate iron stores are important for normal childhood growth and development [1] and IDA may impair cognitive development [2], [3]. Nutritional deficiencies are particularly prevalent in resettled pediatric refugees, who often have significant co-morbidities (including acute chronic infection and/or hemoglobinopathies) which may exacerbate IDA [4].

Hepcidin, a type II acute phase peptide, plays a central role in the regulation of iron homeostasis [5], [6]. Abnormal hepcidin levels have been linked to anemia of chronic disease (ACD) and hemochromatosis [6]. Hepcidin production is driven by pro-inflammatory cytokines, particularly interleukin-6 (IL-6) [7]. It has been proposed that the refractory IDA associated with *Helicobacter pylori* infection may be mediated by inflammation-driven hepcidin production [8]. There are few *in vivo* human data, particularly in children, to substantiate these putative roles of hepcidin in either IDA or in infections.

We explored associations between IDA, urinary hepcidin and cytokine levels in a pediatric population with a high prevalence of infections, including *H. pylori* and *Plasmodium falciparum*. The primary aim was to assess hepcidin levels in refugee children in whom a high prevalence of IDA and *H. pylori* infection was expected. The secondary aim was to investigate the effect of co-morbid infections on (i) ID and IDA, (ii) urinary hepcidin levels and (iii) cytokine levels. We hypothesized that children with *H. pylori* infection would have a higher prevalence of IDA, higher urinary hepcidin levels and increased inflammatory cytokines, particularly of IL-6.

## Methods

### Study population and design

A cross-sectional study was conducted at the initial health assessment unit responsible for screening the majority of humanitarian refugees resettled in Western Australia (WA) [9]. The health assessment takes place over two visits, one week apart, at a median time of six weeks post-resettlement (Dr A Thambiran, Medical Director, Migrant Health Unit (MHU), personal communication, 2007). African children (<16 years) were assessed between February and November 2006 and consecutively recruited, with informed consent obtained in the presence of trained interpreters, as appropriate. Ethical approval was obtained from the Women and Children's Ethics Committee, Princess Margaret Hospital for Children, Perth, Australia (Reference #: EC06-04.13).

Data on age, gender, ethnicity, pre-migration and recent drug administration, the presence of gastrointestinal symptoms (in children ≥2 years) and details of menarche for pubertal girls were obtained. Blood and urine samples were obtained at the first visit and fecal samples at the second, as part of routine clinical care [10]. Children were excluded if they had received antibiotics or specific treatment for *H. pylori* in the preceding month, had a diagnosis of immunodeficiency or active tuberculosis, or if *H. pylori* fecal antigen testing was not performed.

### Hematological analyses

Complete blood count, blood film, iron and hemoglobinopathy studies (HPLC and genetic analyses) were performed on each child at the initial visit. Anemia was defined as a hemoglobin concentration less than age and gender-adjusted norms used in our pediatric population (PathWest Laboratories, Nedlands, Perth, WA) [11]. The reference intervals used in this study are comparable to those of the *World Health Organization (WHO) Guidelines for anemia and iron deficiency* [12]. As ferritin, an acute phase reactant, may be elevated during co-morbid infection(s), iron deficiency (ID) was defined as ≥2 abnormal age-corrected iron parameters (iron, ferritin, transferrin and transferrin saturation). IDA was defined as concurrent ID and anemia.

### Urinary hepcidin analysis

Freshly voided urine samples were collected and 10 mL aliquots were centrifuged at 5000 rpm for 5 minutes and frozen at −80°C for batch analyses at the Department of Clinical Chemistry, Radboud University, Nijmegen Medical Centre, The Netherlands. Urinary hepcidin measurements of the bioactive hepcidin-25 isoform were performed by surface enhanced laser desorption ionization – time of flight mass spectrometry (SELDI-TOF MS), using a mass spectrometry method updated from Kemna et al [13]. A synthetic hepcidin-24 peptide (Peptide international Inc, Louisville, KY, USA) was used as an internal standard [14]. After dissolving the lyophilized hepcidin-24 peptide in distilled water (0.5 µM), 5 µL of the solution was added as an internal standard to 495 µL urine sample (5 nmol), immediately followed by application of a 5 µL sample to immobilized copper (Cu^2+^) affinity capture protein chip arrays (IMAC30-Cu^2+^), equilibrated with appropriate buffers according to the manufacturer's instructions (Biorad, Hercules, CA, USA). Samples with hepcidin peak heights >55Int were considered out of linear range and were diluted with blank urine from a patient with juvenile hemochromatosis [15]. The urine hepcidin concentrations were normalized to urine creatinine (Cr) values and are reported as nmol/mmol Cr. Intra-assay or spot-to-spot variation of urinary hepcidin ranged from 6.1% at 3.2 nmol to 7.3% at 1.2 nmol (n = 8). Inter-assay variation ranged from 7.9% at 5 nmol to 10.9% at 1.0 nmol (n = 4). The normal range for adults is 0.01–10.6 nmol/mmol Cr (www.hepcidinanalysis.com). The lower limit of detection lay between 0.003 and 0.037 nmol/mmol Cr.

### Cytokine analyses

Peripheral blood samples for cytokine analyses were obtained from children >2 years of age, centrifuged and serum snap-frozen at −80°C for batch analyses. Assays for IL-6, IL-1β, tumor necrosis factor alpha (TNFα) and interferon gamma (IFNγ) were performed using a commercial assay (High Sensitivity Human Cytokine LINCOplex kit, Millipore, Missouri, USA) according to the manufacturer's instructions.

### Diagnosis of infectious diseases

*H. pylori* infection was diagnosed using monoclonal fecal antigen immunoassay techniques (MFAT) (Amplified IDEIA™ HpStAR™ kits, Dako, Denmark and Oxoid, Australia) in accordance with the manufacturer's instructions and as previously described [16]. *H. pylori* IgG was measured using a commercial assay (Genesis Diagnostics HpG Screen ELISA, Cambridgeshire, England) [17]. Helminth infection was defined by the presence one or more of the following results: (i) positive serology for schistosomiasis and/or strongyloidiasis, (ii) raised IgE levels (>280 kU/L), (iii) peripheral eosinophilia (≥0.7×10^9^/L) or (iv) positive stool microscopy for ova cysts or parasites of known pathogenic helminths. Fecal microscopy for parasites was performed only if there was serological evidence of helminth activity, peripheral eosinophilia or clinical indications, in accordance with clinical protocols. Latent tuberculosis infection (LTBI) was defined as children with a positive QuantiFERON-Gold™ result (Cellestis, Carnegie, Australia), normal chest radiographs and an absence of clinical symptoms suggestive of active TB. All children were screened for malaria in WA with single thick and thin blood films and rapid immunochromatographic testing for *P. falciparum* malaria (Binax NOW®, Portland, USA), irrespective of symptoms or pre-migration anti-malarial treatment.

### Statistical analyses

All data were analyzed using SPSS version 14.0 for Windows (2005 Chicago, Illinois, USA). Continuous variables were transformed where necessary and compared using the independent t-test or Mann-Whitney (MW) test as appropriate. Associations between categorical variables were initially analyzed using Pearson chi-squared or Fisher's exact tests. Log-transformation (natural logarithm) of ferritin and urinary hepcidin was required to normalize the distribution of these variables for regression analyses. Logistic regression models were developed to analyze associations with ID and IDA using clinical and demographic factors as independent variables and adjusting for age and gender. Linear regression models were used to evaluate the effect of age- and gender-adjusted independent variables on log-hepcidin levels as a continuous dependent variable. Statistical significance was set at the 5% level and 2-sided p-values were calculated.

## Results

### Characteristics of the study population

In total, 198 children were recruited with 17 subsequently excluded (5 had received recent antibiotics, 11 did not have MFAT performed and 1 child did not have hematological assessment). Of the 181 children included in subsequent analyses, 93 were male (51.4%). The mean age was 8.0 years (standard deviation (SD) 4.3 years). There were six ethnic groups with Sudanese (33.7%), Burundian (28.7%) and Liberian (12.7%) children predominating. Ten girls had attained menarche at a mean age of 14.2 years (SD 1.0 years). Approximately half the children (84/162, 51.8%) had no gastrointestinal symptoms at the time of interview. There was no difference in symptoms in the prevalence of symptoms in children with and without *H. pylori* or helminth infections (*H. pylori* infected: 69/137 (50.4%) asymptomatic versus 68/137 (49.6%) symptomatic, p = 0.292 and helminth infected: 40/75 (53.3%) asymptomatic versus 35/75 (46.7%), p = 0.274 respectively).

### Hematologic parameters and iron status

Hematologic characteristics of the study population are summarized in Table 1. Fifty six children (30.9%) were anemic and 35 (19.3%) had ID (Table 2). IDA was present in 25 of 181 children (13.8%) who were younger than non-anemic children (6.3 years, SD 5.0 versus 8.3 years, SD 4.2, p = 0.033). There was no gender difference in the prevalence of IDA. A third of children (50/181) had a hemoglobinopathy with alpha thalassemia trait (single gene deletion) (20/50), sickle cell anemia (HbS) trait (15/50) and alpha thalassemia trait (single gene deletion)/HbS trait (7/50) the most common findings. No child was homozygous for thalassemia, HbS or double heterozygotes for β^0^ thalassemia/HbS. Hemoglobinopathies were not associated with ID, IDA or urinary hepcidin levels (Tables 3 and 4). None of the girls who had attained menarche had IDA.

**Table 1:** Characteristics of study population (n = 181 African refugee children).

VARIABLE	Estimate*
**Age (years)**	8.0±4.3
**Gender (male) (%)**	93/181 (51.4)
**Breastfeeding (%)**	16/181 (8.8)
**Attained menarche (%)**	10/88 (11.4)
**Hemoglobin (g/L)**	118.2±14.7
**Mean cell volume(fL) (IQR)**	80.0 (74.0–83.5)
**Iron (**μ**mol/L)**	11.8±5.2
**Ferritin (**μ**g/L) (IQR)**	32.0 (18.0–47.0)
**Transferrin (**μ**mol/L)** * **(IQR)**	36.0 (33.5–40.0)
**Transferrin saturation (%)**	16.6±7.9
**Urinary hepcidin** ** **(nmol/mmol Cr) (IQR)**	2.2 (0.8–6.2)
**IL-1β Level** † **(pg/mL) (IQR)**	4.5 (2.8–5.4)
**IL-6 Level** ‡ **(pg/mL) (IQR)**	14.7 (11.0–26.2)
**TNFα Level** § **(pg/mL) (IQR)**	9.7 (7.5–11.8)
**IFNγ Level** ‡ **(pg/mL) (IQR)**	22.8 (9.1–36.0)
**Anemia (%)**	56/181 (30.9)
**Iron deficiency (%)**	35/181 (19.3)
**Iron deficiency anemia (%)**	24/181 (13.3)

SD: standard deviation; IQR: interquartile range; %: percentage.

* values represent mean±SD, median and IQR or proportion.

** n = 147.

† n = 138.

‡ n = 139.

§ n = 130.

**Table 2:** Comparison of hematological parameters by presence versus absence of (i) iron deficiency* and (ii) iron deficiency anemia.

VARIABLE	IRON DEFICIENCY			IRON DEFICIENCY ANEMIA		
	ID (n = 35)	NON-ID (n = 146)	p-VALUE	IDA (n = 25)	NON-IDA (n = 156)	p-VALUE
**Hemoglobin (g/L)±SD**	106.4±18.8	121.1±120.2	**<0.001**	97.9±12.8	121.5±12.2	**<0.001**
**Mean cell volume (fL) (IQR)**	73.0 (62.0–79.0)	80.0 (76.0–84.0)	**<0.001**	69.0 (60.5–74.0)	80.0 (76.0–84.0)	**<0.001**
**Iron (**μ**moL/L)±SD**	5.7±2.4	13.3±4.5	**<0.001**	5.2±2.0	12.9±4.7	**<0.001**
**Ferritin (**μ**g/L) (IQR)**	13.0 (7.0–22.0)	35.0 (22.7–51.2)	**<0.001**	9.0 (5.0–16.5)	35.0 (22.0–49.5)	**<0.001**
**Transferrin (**μ**moL/L) (IQR)**	41.0 (37.0–48.0)	36.0 (33.0–39.0)	**<0.001**	47.0 (39.5–49.5)	36.0 (33.0–39.0)	**<0.001**
**Transferrin saturation (%)±SD**	7.0±3.6	18.8±6.8	**<0.001**	6.1±3.1	18.2±7.1	**<0.001**
**Hepcidin (nmol/mmol Cr) (IQR)**	0.3 (0.07–4.0)	2.9 (0.9–6.4)	**0.004**	0.1 (0.05–0.6)	3.0 (0.9–6.7)	**<0.001**

* ID: iron deficiency defined as the presence of ≥2 abnormal/low iron parameters (age and gender adjusted).

IDA: iron deficiency anemia defined as the presence of anemia and iron deficiency; SD: standard deviation; IQR: interquartile range; %: percent. Parameter estimates represent mean±SD or median and IQR.

**Table 3:** Odds ratios for clinical and hematological predictors of (i) iron deficiency* and (ii) iron deficiency anemia.

VARIABLE	IRON DEFICIENCY				IRON DEFICIENCY ANEMIA			
	Number (ID present; %)	Odds ratio	95% CI	*p*-value	Number (IDA present; %)	Odds ratio	95% CI	*p*-value
**Age (years)**	181 (35; 19.3)	0.926	0.849–1.011	0.085	181 (25; 13.8)	0.896	0.809–0.993	**0.036**
**Gender**								
**Female**	88 (11; 12.5)	reference			88 (9; 10.2)	reference		
**Male**	93 (24; 25.8)	2.435	1.112–5.333	**0.026**	93 (16; 17.2)	1.824	0.760–4.375	0.178
**Log-hepcidin**	147 (23; 15.6)	0.640	0.484–0.846	**0.002**	147 (15; 10.2)	0.373	0.242–0.573	**<0.001**
**IL-6 level (pg/mL)** †	139 (23; 16.5)	1.022	0.997–1.048	0.082	139 (15; 10.8)	1.003	0.972–1.034	0.863
**IL-1β level (pg/mL)** †	138 (22; 15.9)	1.048	0.863–1.271	0.637	138 (14; 10.1)	1.084	0.869–1.353	0.475
**TNFα level (pg/mL)** †	130 (23; 17.7)	1.009	0.923–1.103	0.847	130 (15; 11.5)	1.058	0.969–1.154	0.208
**IFNγ level (pg/mL)** †	139 (23; 16.5)	1.020	0.998–1.043	0.075	139 (15; 10.8)	1.022	0.997–1.047	0.087
**Hemoglobinopathy present** †								
**No**	127 (26; 20.5)	reference			127 (18; 14.2)	reference		
**Yes**	54 (9; 16.7)	0.699	0.293–1.664	0.418	54 (7; 13.0)	0.896	0.332–2.310	0.789
***H. pylori*** ** infection** †								
**No**	33 (7; 21.2)	reference			33 (6; 18.2)	reference		
**Yes**	148 (28; 18.9)	0.992	0.367–2.683	0.987	148 (19; 12.8)	0.830	0.285–2.417	0.732
**Helminth infection** †								
**No**	105 (21;20.0)	reference			105 (15; 14.3)	reference		
**Yes**	76 (14;18.4)	1.195	0.500–2.855	0.688	76 (10; 13.2)	1.464	0.537–3.989	0.457
**Malaria infection** †								
**No**	165 (32; 19.4)	reference			165 (22; 13.3)	reference		
**Yes**	16 (3; 18.8)	1.026	0.265–3.976	0.970	16 (3; 18.8)	1.711	0.431–6.795	0.445
**Predeparture albdendazole** †								
**No**	38 (11; 28.9)	reference			38 (7; 18.4)	reference		
**Yes**	143 (24; 16.8)	0.527	0.221–1.258	0.149	143 (18; 12.6)	0.281	0.280–2.061	0.588

* ID: iron deficiency defined as ≥2 abnormal iron parameters.

%: percent; 95% CI: 95 percent confidence interval.

† age- and gender-adjusted variable.

**Table 4:** Adjusted linear regression* analyses for log-hepcidin levels.

	Beta	95% CI	SE	*p*-value
**Age (years)**	−0.039	−0.109, 0.030	0.350	0.266
**Gender (male = 1)**	0.454	−0.071, 0.979	0.266	0.090
***Hematologic parameters***				
**Hemoglobin (g/L)**	0.042	0.022, 0.063	0.010	**<0.001**
**Mean cell volume (fL)**	0.110	0.070, 0.150	0.027	**<0.001**
**Iron (**μ**Mol/L)**	0.064	0.011, 0.117	0.027	**0.018**
**Log-ferritin**	1.295	1.022, 1.569	0.138	**<0.001**
**Transferrin (**μ**Mol/L)**	−0.117	−0.151, −0.082	0.018	**<0.001**
**Transferrin saturation (%)**	0.063	0.030, 0.096	0.017	**<0.001**
**IL-6 level (pg/mL)**	−0.005	−0.024, 0.014	0.010	0.587
**IL-1β level (pg/mL)**	0.019	−0.103, 0.142	0.062	0.754
**TNFα level (pg/mL)**	−0.009	−0.068, 0.051	0.030	0.770
**IFNγ level (pg/mL)**	−0.005	−0.020, 0.010	0.007	0.510
***Dichotomous clinical parameters (presence = 1; absence = 0)***				
**Anemia**	−1.282	−1.838, −0.725	0.282	**<0.001**
**Iron deficiency**	−1.361	−2.054, −0.667	0.351	**<0.001**
**Iron deficiency anemia**	−2.555	−3.323, −1.787	0.388	**<0.001**
**Hemoglobinopathy present**	−0.165	−0.753, 0.423	0.297	0.579
***H. pylori*** ** infection** †	−0.214	−0.970, 0.542	0.382	0.576
***H. pylori*** ** seropostivity**	0.082	−0.508, 0.673	0.298	0.783
**Helminth infection**	0.072	−0.500, 0.644	0.289	0.803
**Malaria infection**	0.497	−0.368, 1.363	0.438	0.258
**Predeparture albendazole**	0.281	−0.376, 0.939	0.333	0.399

* age and gender adjusted.

† *H. pylori* infection diagnosed using monoclonal fecal antigen methods.

Beta: Beta co-efficient; IDA: iron deficiency anemia; 95% CI: 95percent confidence interval; SE: standard error; %: percentage.

Logistic regression analyses were performed to assess the relationship between a range of predictive variables and the presence of ID and IDA (Table 3). In the adjusted model, log-hepcidin was inversely related to ID (odds ratio (OR) 0.64, 95% CI 0.48–0.85, p = 0.002) whereas male gender significantly increased the odds of ID (OR 3.25, 95% CI 1.10–9.62, p = 0.034). For those with IDA, univariate analysis indicated that age (in years) and log-hepcidin were inversely associated. In the age- and gender-adjusted model, only lower log-hepcidin levels were significantly associated with IDA (OR 0.37, 95% CI 0.24–0.57, p<0.001).

### Urinary hepcidin analyses

Urinary hepcidin assays were performed in 147 children. The median hepcidin level was 2.23 nmol/mmol Cr (interquartile range (IQR) 0.83–6.17). Children with ID and IDA had significantly lower hepcidin levels compared to those with normal hematologic parameters (Table 2). Adjusted linear regression analyses demonstrated that log-hepcidin was positively associated with hemoglobin, mean cell volume (MCV), iron, log-ferritin and transferrin saturation levels and negatively associated with transferrin (Table 4). The presence of anemia, ID and IDA also were negatively associated with log-hepcidin levels. Log-ferritin and log-hepcidin levels were significantly correlated (Pearson r = 0.587, p<0.001).

### Infectious diseases

Pre-migration administration of albendazole (an anti-helminthic agent) was documented in 143 children (79.0%), with the remainder receiving empiric albendazole at the first health assessment visit. The timing of albendazole administration did not influence ID, IDA or hepcidin levels (Tables 3 and 4). Helminth infections were diagnosed in 76/181 children (42.0%). Eosinophilia was present in 24 children; 12 had positive serology/fecal microscopy and elevated IgE levels, 5 had normal IgE levels with positive serology, 5 had elevated IgE levels with negative serology and only 2 had isolated eosinophilia (but had received pre-departure albendazole therapy). Elevated IgE levels were found in 63/181 children (34.8%). Eosinophil and IgE levels were significantly higher in children with helminth infections (but not *H. pylori* infection) (Table 5). *P. falciparum* malaria was detected in 16 children (8.8%) and 11 children (6.1%) had LTBI. No child with LTBI had ID or IDA; LTBI was therefore not included as a covariate in logistic regression analyses.

**Table 5:** Comparison of hematological parameters and serum cytokine levels by presence versus absence of (i) *H. pylori* infection* and (ii) helminth infection.

VARIABLE	*H. pylori* infection			Helminth infection		
	Positive†	Negative‡	p-value	Positive§	NegativeØ	p-value
**Hemoglobin (g/L)±SD**	119.6±14.9	112.2±12.3	0.010	120.9±14.2	116.3±14.9	0.036
**Mean cell volume (fL) (IQR)**	80.0 (76.0–84.0)	75.0 (70.5–80.0)	0.001	80.0 (76.0–84.0)	79.0 (73.0–83.0)	0.065
**Iron (**μ**moL/L)±SD**	11.9±5.3	11.6±4.7	0.814	12.4±5.2	11.4±5.1	0.244
**Ferritin (**μ**g/L) (IQR)**	32.0 (18.0–48.0)	32.0 (17.0–43.0)	0.525	37.5 (22.0–51.7)	28.0 (15.0–41.0)	0.002
**Transferrin (**μ**moL/L) (IQR)**	36.0 (33.0–41.0)	37.0 (35.0–39.5)	0.503	36.0 (33.0–40.0)	37.0 (34.0–40.5)	0.294
**Transferrin saturation (%)±SD**	16.7±8.1	15.9±6.8	0.586	17.4±7.7	16.0±8.0	0.241
**Hepcidin (nmol/mmol Cr) (IQR)**	2.2 (0.8–6.2)	3.8 (0.8–5.8)	0.598	2.4 (0.8–5.4)	2.2 (0.8–7.2)	0.669
**Eosinophil level (×10^9^/L) (IQR)**	0.2 (0.1–0.4)	0.1 (0.05–0.3)	0.085	0.3 (0.1–0.7)	0.1 (0.07–0.2)	<0.001
**IgE (kU/L) (IQR)**	160.0 (53.5–467.0)	135.5 (29.0–433.2)	0.300	583.5 (312.7–1282.5)	60.0 (27.0–134.0)	<0.001
**IL-6 level (pg/mL) (IQR)**	14.5 (10.8–24.3)	16.1 (11.4–40.2)	0.262	15.8 (11.3–22.0)	14.5 (10.8–36.4)	0.771
**IL-1β level (pg/mL) (IQR)**	4.5 (2.9–5.3)	4.8 (2.3–6.5)	0.507	4.8 (3.0–5.6)	4.3 (2.4–5.4)	0.377
**TNFα level (pg/mL) (IQR)**	9.5 (7.6–11.7)	11.7 (6.9–13.5)	0.163	10.5 (7.6–12.9)	9.0 (7.2–11.7)	0.183
**IFNγ level (pg/mL) (IQR)**	24.8 (8.3–36.4)	17.5 (11.6–33.9)	0.636	23.7 (10.9–40.7)	21.5 (7.2–34.8)	0.299

* *H. pylori* infection diagnosed using monoclonal fecal antigen methods.

† n = 148 (hemoglobin, MCV, eosinophil, iron, ferritin, transferrin, transferrin saturation), 145 (IgE), 125 (hepcidin), 117 (IL-6, IFNγ), 116 (IL-1β), and 110 (TNFα).

‡ n = 33 (hemoglobin, MCV, iron, ferritin, transferrin, transferrin saturation, eosinophil), 32 (IgE), 22 (hepcidin), 22 (IL-6, IL-1β, IFNγ) and 20 (TNFα).

§ n = 76 (hemoglobin, MCV, iron, ferritin, transferrin, transferrin saturation, eosinophil, IgE), 69 (hepcidin), 58 (IL-6, IFNγ), 57 (IL-1β, TNFα).

Ø n = 105 (hemoglobin, MCV, eosinophil, iron, ferritin, transferrin, transferrin saturation), 78 (hepcidin), 102 (IgE), 81 (IL-6, IL-1β, IFNγ) and 73 (TNFα).

SD: standard deviation; IQR: interquartile range; %: percent. Parameter estimates represent mean±SD or median and IQR.

There was a high prevalence of *H. pylori* infection in this cohort (148/181; 81.8%) [18]. Children with *H. pylori* infection were significantly older (8.5 years (SD 4.2) versus 5.8 years (SD 4.3), p = 0.001) and had a higher mean hemoglobin (119.5 g/L (SD 14.9) versus 112.2 g/L (SD 12.3), p = 0.010). Hematologic parameters and cytokine levels for children with and without *H. pylori* and helminth infections are shown in Table 5.

Ferritin levels were significantly higher in those children with helminth (n = 76) and malaria infections (n = 16), but not in those with *H. pylori* infection (n = 148). The median ferritin levels for helminth, malaria and *H. pylori* infected versus non-infected children were, respectively; 37.5 µg/L (IQR 22.0–51.7) versus 28.0 µg/L (IQR 15.0–41.0), MW p = 0.002; 39 µg/L (IQR 25.0–55.7) versus 31 µg/L (IQR 16.0–46.5), MW p = 0.046; and 32.0 µg/L (IQR 18.0–48.0) versus 32.0 µg/L (IQR 17.0–43.0), MW p = 0.525.

No co-morbid infections were associated with ID, IDA or hepcidin on regression analyses (Tables 3 and 4). Urinary hepcidin levels were not significantly different between children with (n = 69) or without (n = 78) helminth infections MW p = 0.669) nor in children with (n = 125) or without (n = 15) *H. pylori* infection (MW p = 0.598). Median hepcidin levels in children with helminth and *H. pylori* infection were 2.42 nmol/mmol Cr (IQR 0.84–5.41) and 2.17 nmol/mmol Cr (IQR 0.84–6.20), respectively (Table 5). No differences in hepcidin levels were demonstrated in children who were *H. pylori* seropositive (n = 78) compared to those who were seronegative (n = 62) (data not shown). In children with malaria (n = 16), median hepcidin levels were higher (4.69 nmol/mmol Cr (IQR 0.96–9.38) versus 2.19 nmol/mmol Cr (IQR 0.81–5.99) but this was not significant (MW p = 0.209). Eight children had a repeat urinary hepcidin assay one month post malaria treatment (median hepcidin 1.65 nmol/mmol Cr (IQR 0.48–3.04), with the reduction in hepcidin levels trending downwards (Wilcoxon rank sum test p = 0.051).

### Cytokine analyses

Serum cytokines were measured in 139 children (Table 1). There were no significant associations between ID, IDA or hepcidin levels and circulating IL-1β, IL-6, TNFα or IFNγ (Tables 3 and 4). IL-6 levels did not differ significantly between children with or without *H. pylori* infection diagnosed by MFAT (*H. pylori* infected 14.5 pg/mL (IQR 10.8–24.3) versus uninfected 16.1 pg/mL (IQR11.4–40.2), MW p = 0.262) (Table 5) or by serology (data not shown).

## Discussion

This is the first *in vivo* study to explore the associations between hepcidin, iron status, co-morbid infections and cytokine levels in children. Urinary hepcidin levels were significantly lower in children with ID and IDA. There was no relationship between hepcidin and serum cytokine levels, *H. pylori* or helminth infections. Our results support a feedback mechanism between IDA and/or low ferritin and hepcidin secretion. The effect of transferrin on hepcidin is likely mediated by low transferrin-bound iron and/or anemia [6].

### Hepcidin and iron deficiency parameters

ID and IDA are common in refugee children with a prevalence of IDA similar to other non-Caucasian ethnic groups [19], [20] and higher than that in Caucasian Australian children [21]. Nutritional deficiencies are highly prevalent in paediatric refugees resettled in Australia [4], partly due to lack of dietary iron, prolonged breastfeeding and/or delayed introduction of appropriate solid foods and excessive intake of cow's milk. All children were United Nations High Commissioner of Refugees (UNHCR) designated refugees, and were likely to have experienced nutritional and socioeconomic deprivation. Additional nutritional data were not collected as part of this study.

Many studies and guidelines define iron stores (and hence ID) by a single low ferritin level [1], [12]. Ferritin is an acute phase reactant and thus a poor measure of iron status in populations in whom infections are prevalent. The WHO defines ID as a ferritin of <30 µg/L in the presence of infection and/or a combination of other iron parameters to increase the specificity of ID detection [12]. In this study, the broader definition of ID took account of the likely confounding effect of common co-morbid infections on ferritin levels. We found that children with helminth and malaria infections (but not *H. pylori*) had higher ferritin levels than uninfected children. Children in this cohort with replete iron stores (based on our ID definition) had median ferritin levels above 30 µg/L, in keeping with the WHO guidelines. Although iron levels may be subject to diurnal variation, [22] samples were collected during a relatively narrow time period (late morning to early afternoon) and iron levels were not interpreted in isolation, rather combined with other iron parameters.

Hepcidin is produced by hepatocytes and is rapidly cleared from the circulation [23]. Urinary hepcidin levels correlate well with hepatic hepcidin mRNA [24]. Three hepcidin isoforms (hepcidin-20, -22 and -25) are excreted in urine with hepcidin-25 and -20 also found in serum [13], 23, 25. Only hepcidin-25 has a dominant role in iron regulation and is measured in mass spectrometry analyses [13], [25]. A strength of this study was the use of an internal standard, which allowed quantitative assessment of urinary hepcidin levels, in contrast to previously reported mass-spectrometry based. Urine testing was chosen in preference to serum assays as (i) it is less affected by diurnal variation, [13] and (ii) the non-invasive nature of sampling (which was important given the high level of past trauma in refugee children).

The role of hepcidin in iron metabolism and hepcidin regulation is increasingly being defined [5], [6], [26], [27], [28]. However, the majority of data are from *in vitro* or murine models, and *in vivo* human studies are largely of adult patients with hemaochromatosis, [29], [30] hemoglobinopathies, [31], [32] chronic renal failure [33] or infectious/inflammatory disease, [29], [34], [35], [36] where data cannot be extrapolated to children and/or IDA. ACD in childhood is usually related to chronic infection (e.g. chronic osteomyelitis, tuberculosis), or chronic inflammatory conditions including systemic juvenile arthritis, systemic lupus erythematosus, inflammatory bowel disease or chronic renal disease [37]. As occurs in adults, the low serum iron levels seen in children with ACD are accompanied by iron accumulation in tissue macrophages and is thought to be driven by pro-inflammatory cytokines (IL-1 and IL-6) which up-regulate hepcidin production [37].

In iron loading anemia's, such as thalassemia and sickle cell anemia, hepcidin levels are related to iron burden, but are inversely related to the inefficiency and extent of erythropoiesis[5], [6], [31], [3] . We demonstrated that hepcidin levels were significantly lower in African children with IDA and correlated with ferritin levels, but are unaffected by those with hemoglobinopathy traits.

### Inter-relationship between hepcidin, IDA and *H. pylori* infection

Hepcidin expression is induced by iron stores, and inflammation (particularly IL-6) [6], [24], [28]. It is also down-regulated by hypoxia and anemia, erythropoiesis [38]. Mediators such as hemojuvelin and bone morphogenetic proteins are central to hepcidin signaling pathways [39], [40]. In humans, functional mutations of transmembrane serine protease 6 gene (TMPRSS6), an inhibitor of hepcidin expression, result in inappropriately high hepcidin production and severe and iron-refractory IDA [41].

IDA is an extra-gastrointestinal manifestation of *H. pylori* infection, [42] but data on *H. pylori*-induced IDA are inconsistent [43], [44], [45], [46]. Potential mechanisms of IDA in *H. pylori* infection include sequestration of iron by the bacteria by various binding proteins and transporters (including lactoferrin [47] and FeoB [48]), increased gastrointestinal blood loss and/or reduced iron absorption secondary to chronic gastritis [43]. Our findings suggest that such a phenomenon is uncommon in African children who have a high prevalence of both IDA and *H. pylori* infection, but show no relationship between the two diagnoses. We found that anemia was not increased in those with *H. pylori* infection, which also showed no relationship with hepcidin, and thus our data do not support the proposal that hepcidin is the primary mechanism of *H. pylori*-induced anemia [8].

The chronicity of *H. pylori* infection may be important, in particular the onset of gastritis and/or development of complications such as peptic ulceration, which cannot be identified by MFAT [44], [49]. However it was considered unethical and impractical to perform routine endoscopy on asymptomatic children and we cannot comment further on the relationship between the severity of gastritis and IDA [46], [50]. Moreover, *H. pylori* infection in children, in contrast to adults, results in a pre-dominantly regulatory T-cell response in the gastric mucosa, with local production of the counter-inflammatory cytokines transforming growth factor and IL-10 [51]. The lack of association between hepcidin and levels of circulating cytokines, measured by a high-sensitivity assay, suggest that *H. pylori*-induced inflammation does not influence iron status through increased hepcidin production in childhood. It remains unclear whether a minority of *H. pylori*-infected children develop more marked gastric inflammation, leading to increased pro-inflammatory cytokines, increased hepcidin production and subsequent refractory IDA and/or ACD. However, it is unlikely that this is a widespread mechanism of *H. pylori*-induced anemia in children, as previously proposed [8].

### Hepcidin and other markers of infection

The relationship between infection, inflammation and hepcidin (which has *in vitro* antimicrobial activity) [23] is largely unexplored in humans. In response to inflammation, hepcidin down-regulates duodenal enterocyte iron absorption and macrophage iron release by binding to ferroportin and inducing its internalization and degradation, [52] thus decreasing extracellular bacterial access to iron. It is postulated that bacterial lipopolysaccharide (LPS) stimulates IL-6 production and thus up-regulates hepcidin release leading to hypoferremia, [7], [52] as reported in human endotoxemia models [36]. We did not find any association between serum cytokines and hepcidin levels, [7], [34] although our data are cross-sectional and cytokines have relatively short half-lives in the circulation, both of which are limitations of this study. In comparison, a report of two cases with Castleman's disease (characterized by chronic IL-6 overproduction) showed reduction in hepcidin secretion following treatment with anti-IL-6 receptor antibodies, suggesting that chronic stimulation and/or inflammation may drive inappropriate hepcidin responses and thus ACD [53], [54].

Malaria infection occurred in a minority, but these children had higher median hepcidin levels than those children with helminth or *H. pylori* infection. This may reflect a more marked and acute inflammatory response in malaria than what is elicited by more chronic infections. Our malaria cohort was small thus limiting conclusions, however we demonstrated a non-significant trend for elevated hepcidin levels to fall following malaria treatment. A recent larger study of hepcidin and malaria is in agreement with our findings [55]. Hepcidin may be up-regulated to prevent parasite access to iron, possibly via the induction of hypoferremia [52]. In a small study of Ghanan children with acute falciparum malaria, log-hepcidin levels were associated with log-parasitemia but not with hemoglobin or anemia [56].

Schistosomiasis, strongyloides, and giardiasis were the main enteric infections identified, but none influenced either IDA or hepcidin levels. *Ancyclostoma duodenale* (hookworm) infection has been shown to increase the risk of IDA, by increasing gastrointestinal blood loss [57]. Historically hookworm infection is prevalent in resettled refugees, [9] however *A. duodenale* was not detected in this cohort probably because of empiric anti-helminthic therapy. It seems unlikely that hookworm will influence hepcidin production, but the effects of anti-helminthic treatment on hepcidin may warrant further investigation.

We found no associations between hepcidin secretion and either circulating cytokine levels or co-morbid gastro-intestinal infections. This may be related to both the chronicity and lack of sustained inflammatory response to these infections as opposed to systemic infections such as falciparum malaria. The low levels of ferritin in *H. pylori* infected children, relative to those in children with malaria, also suggests that chronic infection with *H. pylori* does not result in significant or sustained systemic inflammation. The lack of symptoms in children with *H. pylori* and helminth infections in this cohort may also reflect less microbial load, less severe infections and hence less gastrointestinal and systemic inflammation, with consequently no relationship observed between these infections and cytokine and/or urinary hepcidin levels. In addition, the hepcidin effects on its ferroportin-“receptor” are suggested to be cell- and time specific [58]. Moreover, changes in hepcidin levels during inflammation and infections are accompanied by cytokines that may also directly influence erythropoiesis and intestinal iron uptake [59].

### Conclusion

This is the largest *in vivo* study to date to explore the interaction between inflammation, erythropoiesis, anemia and hepcidin production. African refugee children resettled in Australia have a high prevalence of ID and IDA and a high burden of infection. Children with IDA had strongly down-regulated hepcidin expression but this was not influenced by co-morbid gastrointestinal infections and did not correlate with increased inflammatory cytokines. Urinary hepcidin was influenced positively by ferritin and most other hematologic parameters but negatively correlated with transferrin levels.

These findings require confirmation in studies in other populations. Although infections in animal models and adult humans may be useful in understanding the relationship between inflammation and hepcidin, the data are not readily extrapolated to natural infection in children. The burden of infection falls largely on children in developing countries who often have multiple co-morbidities that potentially modify hepcidin and iron. Longitudinal studies of IDA treatment and hepcidin and cytokine responses, together with investigation of putative intermediate mediators and consideration of co-morbid infections, are required to further understand further the role of hepcidin in iron regulation in children.
